# Delay in diagnosis of oral cancer: a systematic review

**DOI:** 10.4317/medoral.24808

**Published:** 2021-10-27

**Authors:** Andrea Márcia da Cunha Lima, Ingrid Andrade Meira, Maria Sueli Marques Soares, Paulo Rogério Ferreti Bonan, Cláudia Batista Mélo, Carmem Silvia Laureano Dalle Piagge

**Affiliations:** 1 Postgraduate Program in Gerontology. Federal University of Paraíba (UFPB), João Pessoa/PB, Brazil; 2 Department of Prosthodontics and Periodontology. University of Campinas (UNICAMP), Piracicaba/SP, Brazil; 3 Department of Clinical and Social Dentistry. Federal University of Paraíba (UFPB), João Pessoa/PB, Brazil; 4 Department of Restorative Dentistry. Federal University of Paraíba (UFPB), João Pessoa/PB, Brazil

## Abstract

**Background:**

Oral cancer represents a worldwide public health problem, being among the most prevalent, associated with high morbidity and mortality rates. This systematic review aimed to review the causes of the delayed diagnosis of oral cancer mainly in the elderly, in developed and developing countries.

**Material and Methods:**

Search strategy was developed for MEDLINE databases (via PubMed), EMBASE, Web of Science, SCOPUS, and LILACS and for grey literature (Google Scholar, ProQuest and OpenGrey), without language or period restrictions. The risk of bias was assessed using instruments from the Joanna Briggs Institute and the quality of evidence according to the GRADE system.

**Results:**

The search resulted in 14,473 records, of which only 13 met the eligibility criteria. The total sample was 1,705 participants, with a predominance of males. All studies included reported causes of delayed diagnosis of oral cancer related to the patient and five also reported causes related to health professionals. The scarce knowledge of the population was pointed out as the main cause of delayed diagnosis of this cancer. Regarding the risk of bias, ten studies were classified as low risk and three, as moderate risk. The quality of the evidence was very low for the outcome related to delayed diagnosis of oral cancer.

**Conclusions:**

Wide dissemination of information on oral cancer is needed, especially for the elderly, such as its initial signs and symptoms, in developed and developing countries. Further studies should be conducted to better understand the causes of delayed diagnosis of oral cancer in countries with different socioeconomic statuses.

** Key words:**Delayed diagnosis, mouth neoplasms, oral neoplasm, aged.

## Introduction

Oral cancer is among the ten most prevalent types of cancer in the world, associated with high mortality and morbidity rates, representing a worldwide public health problem. This cancer is more frequent in men aged over 40 years and has multifactorial etiology, resulting from the interaction of several risk factors, being the main ones smoking and excessive consumption of alcoholic beverages (1). In the elderly population, the number of cases of oral cancer has grown considerably, due, in part, to the increase in longevity of this population (2). In the next 20 years, the global estimate is an increase of 66.2% in the number of new cases of oral cancer for the elderly population (3). In developed countries, such as the United States of America, France, the United Kingdom, Japan, Italy, and Australia, an increase of 24.9% to 50.5% is estimated for the period 2020 through 2040. In developing countries (China, India, and Brazil), considering this same period, an even greater increase in new cases is projected, ranging from 80.1% to 97.8% (3).

These projections show the importance of knowing the characteristics of oral cancer, enabling the promotion of preventive actions and early diagnosis (4). These actions avoid injuries, minimize complications resulting from treatment with higher chances of cure and provide a higher survival rate to patients (3). However, in most cases, oral cancer is at an advanced stage at the time of diagnosis and, despite technological advances, its treatment is still a great challenge, with survival rates without a significant increase in recent decades, remaining between 50-55 over a five-year period (5).

The stages of tumors are classified according to the Classification System of Malignant Tumors (TNM) recommended by the International Union for Cancer Control (UCC). They receive graduations based on the anatomical extension of the disease, usually from T0 to T4, from N0 to N3, and from M0 to M1. The first one (T), respectively, consider the characteristics of the primary tumor, the second (N) the characteristics of the lymph nodes of the lymphatic drainage chains of the organ in which the tumor is located, and the M the presence or absence of distant metastases. T3 or T4 classifications represent advanced stages of cancer (6), which are frequently found at the time of oral cancer diagnosis (5). Given the above, this study aimed to review the causes of delayed diagnosis of oral cancer, especially in the elderly, in developed and developing countries.

## Material and Methods

- Protocol and register

This review was reported according to the items of the Preferred Reporting Items for Systematic Reviews and Meta-Analyses (PRISMA) (7). A study protocol was developed based on PRISMA-P (8) and registered in PROSPERO (9), under registration number CRD42020214639.

- Eligibility criteria

The acronym PECOS (Population, Exposure, Comparison, Outcome, Study design) was used to formulate the research question of this systematic review, the population was defined as the elderly, the exposure as oral cancer, the primary outcome as causes of delay, and the study design as observational ones. Therefore, the following question was established: "Is there a difference in the causes of the delayed diagnosis of oral cancer mainly in the elderly in developed and developing countries?"

The inclusion criteria consisted of observational studies, without language or period restriction, which evaluated the diagnosis of oral cancer in the elderly. The following exclusion criteria were applied: 1) studies that did not include participants aged 60 years or older; 2) studies that did not evaluate the causes of late diagnosis; 3) studies that did not report data related to the causes of late diagnosis only for oral cancer; 4) studies that performed evaluations only to the treatment of oral cancer. Furthermore, experimental studies, reviews, letters, abstracts, opinion articles, case reports, case series, and book chapters were excluded.

This review was conducted to verify the causes of the delayed diagnosis, especially in the elderly, due to the high incidence of oral cancer in this population and the frequent presence of unfavorable systemic conditions, which hinder the diagnosis, treatment and cure of this disease (2). Given this and the lack of primary studies only with the elderly, this review comprised only those studies that also included elderly participants. The age group characterized for the elderly was over 60 years, because it is still adopted by some developing countries, such as Brazil (10).

Finally, the criteria proposed by the United Nations Development Programme (UNDP) of the United Nations (UN) was adopted to classify developed and developing countries. These criteria are based on the degree of wealth, level of industrialization and development, Gross Domestic Product (GDP), per capita income, and the Human Development Index (HDI), which represents a comparative measure of countries regarding wealth, literacy, education, life expectancy and birth expectancy (11).

- Search strategy

The literature search was conducted by two independent researchers [AMCL and CSLDP], at MEDLINE databases (via PubMed), EMBASE, Web of Science, SCOPUS and LILACS, and in the gray literature (Google Scholar, ProQuest, and OpenGrey).

With the collaboration of a librarian, a search strategy was set up for PubMed and adapted to the other databases. The strategy adopted sought to rescue as many studies as possible related to the subject. For this, the descriptors indexed in the Health Science Descriptors (DeCS) and in the Medical Subject Headings (Mesh Terms) were used, with the Boolean operators AND and OR, as described in Table 1.

**Table 1:**
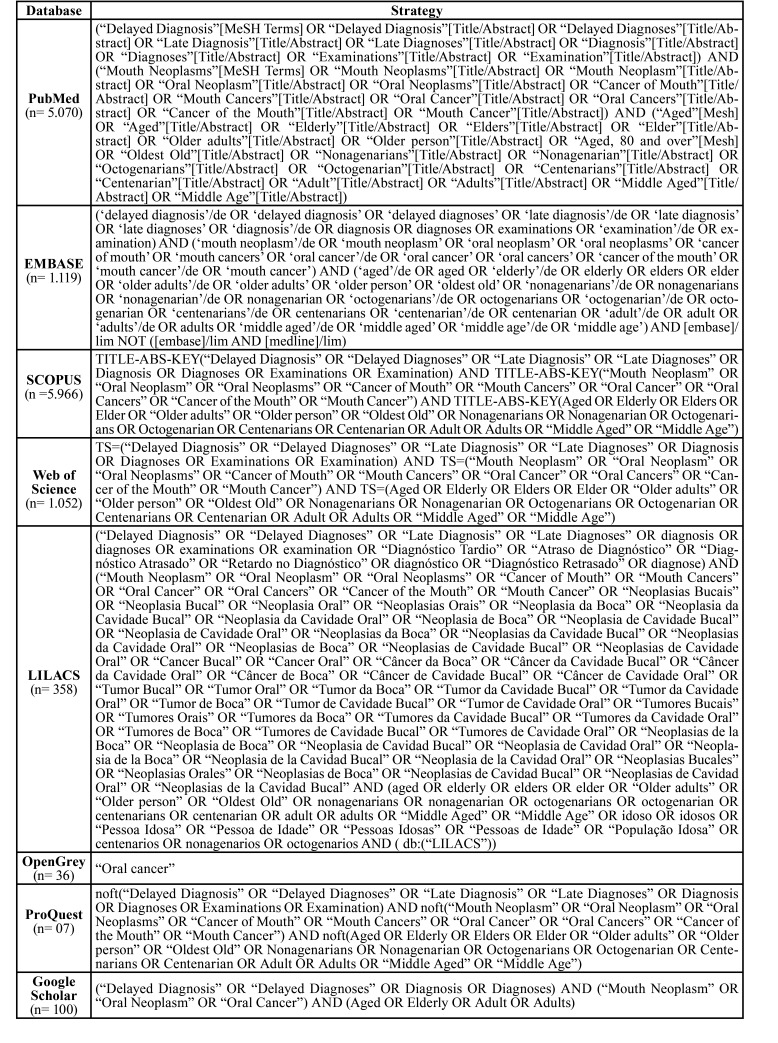
Search strategy performed at the databases until Nov 2020.

- Selection of the studies

The reference manager, Mendeley® Desktop, was used to remove duplicate records and organize primary studies.

The study selection process was carried out in two phases and independently by two reviewers [AMCL and CSLDP]. In the first phase, the two reviewers used Rayyan software (Qatar Computing Research Institute) to exclude studies that did not meet the eligibility criteria (12). In the second phase, after reading the full text and applying the eligibility criteria, a third reviewer [IAM] was consulted for conflict resolution and, after achieving the consensus, the final decision was reached for the studies in disagreement.

- Data extraction and qualitative synthesis

The information of the selected studies was collected by the first reviewer [AMCL] and confirmed by the second reviewer [CSLDP]. The data collected were: author, year of publication, country of study, type of study, general sample, participants’ age, sample loss, form of data collection, and reported causes for the delayed diagnosis of oral cancer.

- Assessment of methodological quality

The methodological quality was evaluated using checklists for analytical cross-sectional and qualitative studies from Joanna Briggs Institute (13,14). Independently, two reviewers [AMCL and PRFB] performed the risk assessment of bias and, for this trial, the following classification was used: "high risk" when the study reached up to 49% of "yes", "moderate risk" scores when the study reached 50% - 69% of "yes" score and "low risk" when the study reached over 70% of "yes" score (15). After the two reviewers checking the trial, the disagreements were discussed and a third reviewer decided [IAM]. Information on bias risk assessment was generated by RevMan 5.4 software (Review Manager 5.4, The Cochrane Collaboration).

The evaluation of methodological heterogeneity among the eligible studies was evaluated regarding the study design, the sample (age, confirmed diagnosis of oral cancer), quality of measurements and results. Finally, the Grading of Recommendations Assessment, Development, and Evaluation (GRADE) approach was used to classify the quality of evidence for the outcome related to the delayed diagnosis of oral cancer, according to the following criteria: risk of bias, inconsistency, indirect evidence, inaccuracy or publication bias. Based on the GRADE criteria, for each serious concern, a level of evidence was lowered and non-randomized clinical studies started with a low classification (16).

## Results

**Figure 1:**
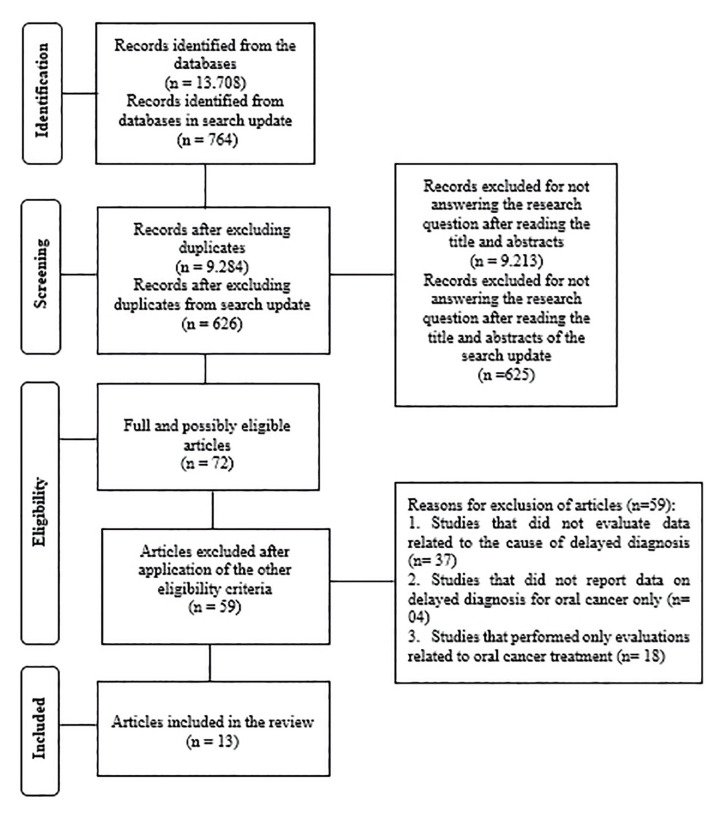
Flowchart of screening process, according to PRISMA statement.

Initially, 13,708 records were identified from the electronic databases and gray literature. After excluding duplicates, 9,284 were analyzed with the reading of titles and abstracts, and 71 articles were selected for full reading. After that, 59 articles were excluded because they did not meet the eligibility criteria, resulting in 12 articles included. A search update was carried out in May 2021, where 764 records were identified. After excluding duplicates, 626 studies were analyzed with the reading of titles and abstracts, selecting 01 article for full reading, which was included, resulting in 13 articles included in this review (Fig. 1).

All studies reported causes of delayed diagnosis of oral cancer related to the patient and 5 also reported causes of delayed diagnosis related to health professionals. Among the causes of delay related to the patient, the lack of knowledge about oral cancer, its risk factors and the signs and symptoms of the disease were reported in all studies. These aspects are due to the difficulty of visualization and identification of the initial lesions, neglect of the signs and initial symptoms due to absence of pain, without impairment of function, leading the participants to consider the symptoms as normal and something secondary. Hope for spontaneous cure, self-medication, fear of diagnosis and coping with the disease, financial restrictions and difficulty in accessing specialized professionals were also reported (6,17-28).

The causes of delay related to professionals were reported in only five studies, four of them conducted in developing countries (17-20) and one in developed country (21). The studies indicated that these causes were related to the difficulty of recognizing the initial lesions and early treatment, leading to incorrect diagnoses, which suggests unawareness of oral cancer among health professionals. The delayed referral of the patient to the specialized service and, consequently, delayed biopsy test were also reported causes.

- Characteristics of the studies

Of the included studies, 13 were observational, four retrospective and two qualitative, published between 1994 and 2020 (Table 2). Of these studies, four were conducted in developed countries (one in Australia, one in Japan, one in Italy, and one in the United Kingdom) and nine in developing countries (two in Brazil, two in China, one in Poland, one in Malaysia, two in Pakistan and one in Thailand). The sample included 1,705 participants aged between 15 and 96 years, in eight studies most participants were aged 60 years and in five studies, this age group was also included, although it did not represent the majority of its participants. There was a higher prevalence of males in the samples of the included studies. In three studies, there was sample loss, 32 participants, four for withdrawal and 28 for incomplete information. The form of data collection that prevailed was the interview through semi-structured questionnaires (eight studies). Clinical records were used by two studies, and other three applied structured questionnaires.

**Table 2:**
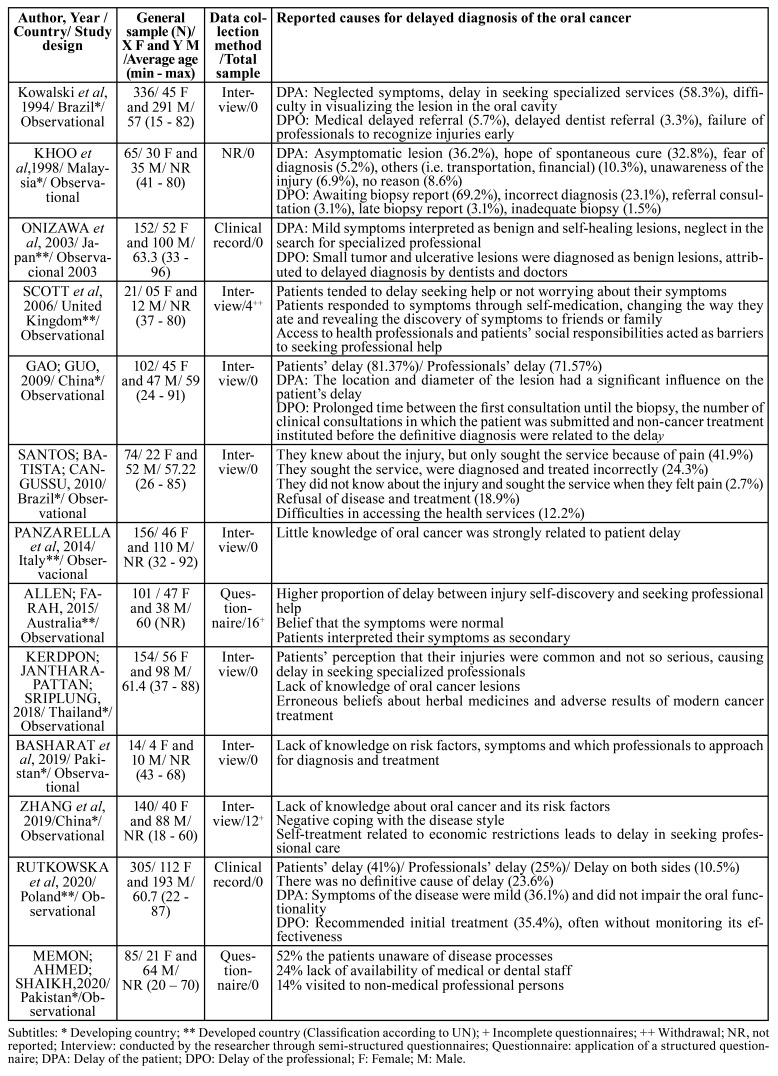
Summary of the descriptive characteristics of the articles included (n = 12).

- Methodological quality

**Figure 2:**
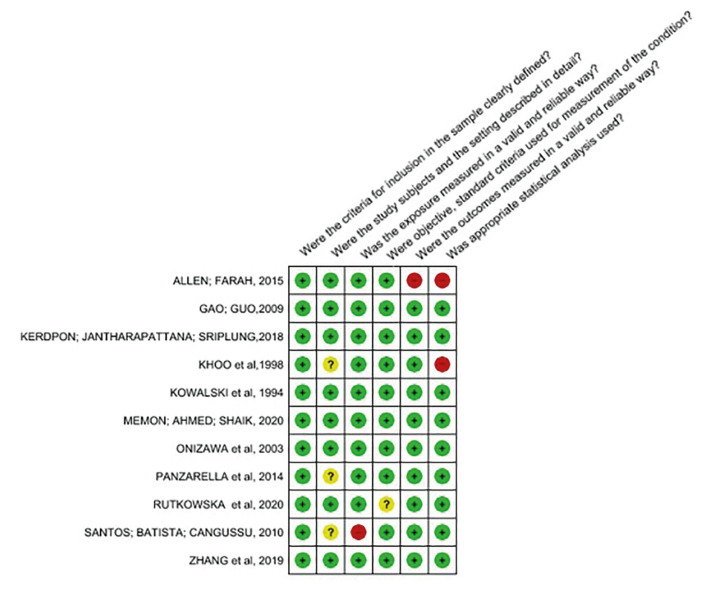
Summary of risk of bias assessed by the Joanna Briggs Institute Critical Assessment Checklist for cross-sectional studies (+) low risk, (-) high risk, (?) Unclear.

**Figure 3:**
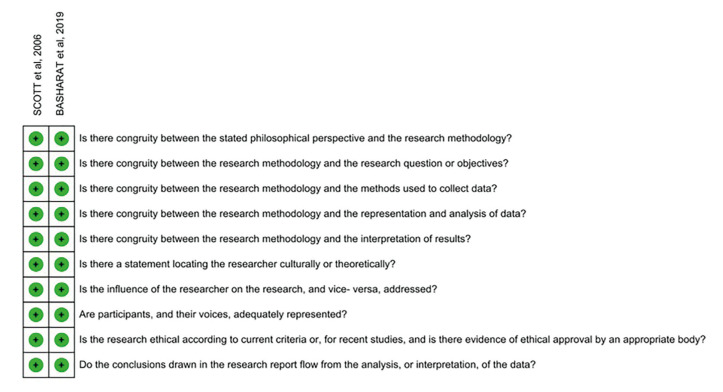
Summary of risk of bias assessed by the Checklist for qualitative research (+) low risk, (-) high risk, (?) Unclear.

Most of the included studies presented a low risk of bias in their evaluation. Of the 11 observational studies, eight presented low risk of bias and three moderate risk (Fig. 2). For these last studies, statistical analysis was the main problem, which was not clearly reported. As for qualitative studies, all of them at a low risk of bias (Fig. 3). Regarding methodological heterogeneity, a great diversity was noted regarding the sample, the form of data collection, and analysis of the results, preventing the quantitative synthesis. Finally, the quality of evidence of the outcome related to the delayed diagnosis of oral cancer was very low (Table 3).

**Table 3:**
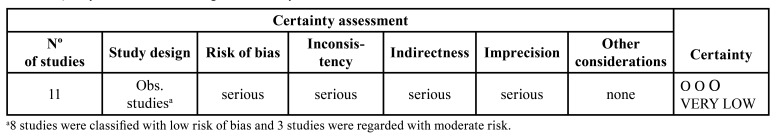
Quality of evidence according to GRADE system.

## Discussion

The results of this systematic review indicate that the population's lack of knowledge about the signs and symptoms of oral cancer and the devaluation of self-care are extremely related to the delayed diagnosis of this cancer.

The sample studies performed their analyzes considering the elderly population along with other age groups, with no reporting of categorizations. A higher prevalence of participants aged ≥ 60 years was identified in the sample, resulting in the hypothesis of greater delay in the diagnosis of oral cancer in this population. Regarding gender in the incidence of oral cancer, the prevalence of males in the sample corroborates other primary studies not included in this review and data from the World Health Organization (6,29). This differs from several studies in the dental field, in which a higher prevalence of females is observed (30).

The studies conducted in developed and developing countries included in this review identified the difficulty of visualization and/or identification of oral lesions and the absence of symptoms of the initial lesions as the main cause in the delayed diagnosis of oral cancer related to the patient (6,17-28). This may be due to the population's little knowledge on oral cancer, its risk factors, and its specific characteristics, leading them to neglect the initial signs and symptoms and, consequently, delay in the search for specialized care. This fact corroborates another study (31) that concluded that the population, even knowing the existence of oral cancer, does not have enough information for the correct diagnosis and prevention of the disease.

In developing countries, fear of diagnosis and negative coping with the disease were also reported as causes of patient delay, probably related to the negative belief of the population that a cancer diagnosis is a death sentence (6,18,24). Still in smaller proportions, hope for spontaneous healing of lesions and self-medication were related to the patient's delayed search for specialized care in developed and developing countries (18,21,22-25). These facts may be associated with difficulties in accessing health services and economic restrictions, which have also been reported as causes of patient delay, not only in studies conducted in developing countries, but also a study included in this review, conducted by Scott *et al*. [2006] (25) in the United Kingdom (6,18,24).

In developed countries, the population has greater access to health services when compared to developing countries, which does not always happen when it comes to a specialized service. As reported in the study by Ligier *et al*. [2016] (32), which concluded that, although the population has universal health coverage, access to specialized care is still restricted to the most economically privileged. As described by those authors (32), the population that presented some type of head and neck cancer rarely attended the dentist, suggesting that the lack of access to dental care is due to financial restrictions. Therefore, socioeconomic conditions may also be associated with the causes of the delayed diagnosis of oral cancer in developed and developing countries.

Although it has not been reported in the studies included in this review, some authors associate the presence of comorbidities with the delay in the diagnosis of oral cancer. This suggests that patients already debilitated, due to the presence of other diseases, especially those dependent or bedridden, ignore or deny new oral symptoms (33). The importance of disseminating knowledge about oral cancer to those who provide care services to these patients is emphasized.

With the severity of the pandemic, anxiety and the population’s fear of becoming infected with COVID-19 has led patients to underestimate the signs and symptoms of other serious diseases, which may lead to delays in the diagnosis of oral cancer (34). Thus, the importance of social media and teledentistry for oral diagnosis during the pandemic period is highlighted, especially in lesions with suspected malignancy (35).

Among the included studies that evaluated the causes of the delayed diagnosis of oral cancer related to professionals in developing countries, the difficulty in recognizing oral cancer lesions early, incorrect diagnosis, and wrong treatments were prevalent. These causes were also reported in the study by Onizawa *et al*. [2003] (21), performed in Japan, in which small tumors and ulcerative lesions were diagnosed as benign lesions, signaling professionals' deficiency of knowledge about oral cancer in both developed and developing countries. Finally, these factors were associated with the delay in the patient’s referral by the doctor or dentist and the performance of the biopsy (6,17-20).

The early diagnosis of oral cancer contributes to the implementation of appropriate treatment in early stages of disease evolution, with less invasive surgeries, faster convalescence and better patient survival (20). On the other hand, the delayed diagnosis increases the care costs of treatment with outpatient or prolonged hospital stay, with main consequences for the most economically disadvantaged (17).

The methodological quality analysis showed that most studies presented a low risk of bias. However, there was a high methodological heterogeneity among the included studies, related to the use of several instruments to analyze the delayed diagnosis and the inclusion of a wide age group, which hindered summarizing and comparing the results, limiting this review. Some studies did not present the clinical staging of tumors through the TNM System, information relevant to the classification of late diagnosis of oral cancer. Moreover, the possible loss of information in retrospective studies and the lack of studies conducted simultaneously in developed and developing countries, adopting the same methodology, hindering comparing more accurately and analyzing the results carefully. The low level of awareness and knowledge about oral cancer by the population and health professionals shows the need for actions aimed at health education with an emphasis on prevention and early diagnosis of oral cancer, to reduce the high mortality and morbidity rates and, consequently, improve the survival of the population, especially the elderly. The causes of oral cancer delayed diagnosis in countries with different socioeconomic statuses need further clarification.

## Conclusions

Despite the lack of methodological standardization, the causes of delayed oral cancer diagnosis were related to the scarce knowledge of patients and health professionals in developed and developing countries. A proposal for a future study is to investigate, in addition to the causes that hinder early diagnosis in the elderly, the reasons for abandonment and/or non-oral cancer treatment.
